# Toxicity and neurophysiological impacts of plant essential oil components on bed bugs (Cimicidae: Hemiptera)

**DOI:** 10.1038/s41598-019-40275-5

**Published:** 2019-03-08

**Authors:** Sudip Gaire, Michael E. Scharf, Ameya D. Gondhalekar

**Affiliations:** Center for Urban and Industrial Pest Management, Department of Entomology, Purdue University, West Lafayette, IN 47907 USA

## Abstract

Bed bugs (*Cimex lectularius* L.) are globally important human parasites. Integrated pest management (IPM) approaches, which include the use of essential oil-based insecticidal compounds, have been proposed for their control. This study aimed to define insecticidal activity and neurophysiological impacts of plant essential oil constituents. The topical and fumigant toxicity of 15 compounds was evaluated against adult male bed bugs. Neurological effects of the 6 most toxicologically active compounds were also determined. In both topical and fumigant bioassays, carvacrol and thymol were the most active compounds. The potency of bifenthrin (a pyrethroid insecticide) in topical bioassays was 72,000 times higher than carvacrol, while vapors of dichlorvos (an organophosphate insecticide) were 445 times more potent than thymol. Spontaneous electrical activity measurements of the bed bug nervous system demonstrated neuroinhibitory effects of carvacrol, thymol and eugenol, whereas linalool produced an excitatory effect. Although citronellic acid and (±)-camphor increased baseline activity of the nervous system their effects were not statistically significant. Bifenthrin also caused neuroexcitation, which is consistent with its known mode of action. These comparative toxicity and neurological impact findings provide new information for formulating effective essential oil-based insecticides for bed bug IPM and conducting mode-of-action studies on individual essential oil components.

## Introduction

Bed bugs (*Cimex lectularius* L.) are economically and medically important global human parasites. They feed on human blood and their bites can worsen psychological disorders, cause sleep deprivation and other health issues such as rashes, itching, allergies, and etc.^1^. The U.S. Center for Disease Control and Prevention (CDC) and the U.S. Environmental Protection Agency (EPA) consider bed bugs as a pest of significant public health importance^2^. A resurgence of bed bugs has occurred over the last 18 years and they continue to spread. One of the primary factors for their resurgence is due to the overuse of synthetic insecticides with similar modes of action, which has led to insecticide resistance development^3–6^. The application of synthetic insecticides within buildings or in indoor environments is also a public health concern due to the toxic effects that can result from prolonged exposure^7–9^.

Integrated pest management (IPM) approaches have been proposed for the effective management of bed bugs. This strategy includes the use of multiple control tactics: resident education, bed bug monitoring using active and passive traps, non-chemical control (removal of infested furniture, heat treatments, use of mattress encasements etc.), along with the use synthetic and essential-oil based insecticides^10–12^. There is also an increased demand from the public for use of efficacious “green” products for urban pest management. Botanical insecticides, including essential oils are considered safe because of their low toxicity to humans and animals^13,14^. Plant-derived essential oils have emerged as a potential alternative option for the management of insect pests^15,16^. Because they pose a minimum risk, essential oil compounds are exempt from full EPA registration (Federal Insecticides, Fungicides, and Rodenticides Act-FIFRA, 40 CFR 152.25)^17^. Some of the drawbacks associated with the use of essential oils for pest control are: (i) short residual life that necessitates frequent applications (ii) high volatility can lead to odor problems, which are sometimes unacceptable to residents, and (iii) field efficacy of these products is generally less documented for different insect pest species^15,16^.

Essential oils are secondary metabolites derived from aromatic plants that are composed of complex mixtures of chemical constituents or components with different functional groups (e.g., phenols, aldehydes, acids, hydrocarbons, etc.)^18^. Recent studies have shown that plant-derived essential oils exhibit contact and fumigant toxicity against field populations of bed bugs^14,19,20^. However, these studies have not characterized the insecticidal activity of major constituents of essential oils against bed bugs. More than a dozen essential oil-based products are available commercially for indoor use, but only two products have been found effective for bed bug control^21^. Therefore, there is a need for conducting comparative baseline toxicity studies with bed bugs using major components or constituents of different plant essential oils (Table S1) that have been shown to be efficacious against urban and agricultural insect pests^22–31^.

There is also a significant knowledge gap regarding the effects of major or active components of essential oils on the insect nervous system^32,33^. The possible target sites for the essential oil components thymol, eugenol, and carvacrol are gamma-amino butyric acid (GABA), octopamine/tyramine and nicotinic acetylcholine (nACh) receptors, respectively^34–37^. Very few studies have documented electrophysiological responses induced by application of essential oil components to the nervous system of insects. Price and Berry^38^ reported that the essential oil components eugenol, geraniol and citral are neurologically active against *Periplaneta americana* and *Blaberus discoidalis*. Similarly, Hertel *et al*.^39^ found the plant essential oil components quassin and cinnamaldehyde to be neurologically active against *P*. *americana*. Recent *in silico* molecular docking studies with major chemical constituents of marigold essential oil (α-terpinolene, piperitone and piperitenone) suggested the neurotransmitter hydrolyzing enzyme acetylcholinesterase as the potential target site in bed bugs^14^.

Given the knowledge gaps associated with the unavailability of comparative toxicity data for individual essential oil constituents against bed bugs, and their impacts on the nervous system, the objectives of this research were (i) to determine topical and fumigant toxicity of fifteen essential oil components against bed bugs and (ii) identify neurological effects caused by the six most effective constituents by performing electrophysiology experiments.

## Results

### Topical toxicity at 24 h

Acetone-diluted essential oil components were applied to the ventral metathorax of adult male bed bugs to determine their topical toxicity. Of the fifteen different components tested, carvacrol and thymol were relatively more toxic with LD_50_ values of 27.5 and 32.5 µg/mg body weight, respectively (Table 1). Both compounds showed similar levels of toxicity based on the relative median potency analysis (Table S2). Similarly, carvacrol and thymol were significantly more toxic than citronellic acid, eugenol, geraniol, α-pinene, R (+)-limonene, linalool, eucalyptol, (−)-terpinen-4-ol, trans-cinnamaldehyde, menthone, (±)-citronellal, (±)-camphor and methyl eugenol (Tables 1 and S2). In the positive control treatment, the pyrethroid insecticide bifenthrin was ~72,000 times more potent than carvacrol with an LD_50_ of 0.000345 µg/mg body weight (Tables 1 and S2).

**Table 1:** Mortality response of adult male bed bugs to topical application of essential oil components and bifenthrin.

Essential oil components	N	LD_50_^a^, µg/mg body weight (95% FL^b^)	Slope ± SE	χ²	d.f.	*P* value
Carvacrol	240	27.5 (25–30.5)a	2.67 ± 0.30	4.82	5	0.507
Thymol	240	32.5 (29.5–35)a	3.32 ± 0.47	2.33	5	0.801
Citronellic acid	270	49 (42–57)b	1.29 ± 0.15	4.05	6	0.669
Eugenol	270	52 (47–57.5)bc	2.20 ± 0.23	6.06	6	0.416
Geraniol	270	64 (55.5–73)bc	1.77 ± 0.19	10.27	6	0.113
α-Pinene	270	70.5 (62–79.5)cd	1.85 ± 0.20	3.89	6	0.690
R (+)-Limonene	240	91.5 (79.5–104)de	1.67 ± 0.20	6.28	5	0.280
Linalool	210	112 (94.5–130.5)e	1.59 ± 0.20	17.31	4	0.002
Eucalyptol	240	132 (118.5–146.5)ef	2.10 ± 0.25	7.67	5	0.175
(−)-Terpinen-4-ol	210	138.5 (125.5–153)efg	2.96 ± 0.44	3.62	4	0.459
trans-Cinnamaldehyde	330	138.5 (116.5–159.5)fg	1.15 ± 0.14	13.73	8	0.192
Menthone	240	165 (136.5–198)gh	1.10 ± 0.14	8.96	5	0.110
(±)-Citronellal	210	240 (211.5–273.5)h	1.81 ± 0.24	10.15	4	0.038
(±)-Camphor	210	515 (454–1121)i	3.27 ± 1.26	0.29	4	0.990
Methyl eugenol	180	560 (350–2655)j	0.76 ± 0.22	4.37	3	0.223
**Positive control**						
Bifenthrin	180	0.000345 (0.0003–0.000405)k	1.73 ± 0.25	0.68	3	0.877

^a^LD_50_ = median lethal dose necessary to kill 50% of individuals. ^b^95% FL = 95% fiducial limits. LD_50_ values with the same letter are not significantly different based on the relative median potency analysis (refer to Table S2 for details). Mortality in control groups was 0%, except in linalool (3.33% mortality). Body weight of a single adult male bed bug used for bioassays was approximately 2 mg.

### Fumigant toxicity at 24 h

Adult male bed bugs were exposed to vapors of essential oil components in sealed mason jars (volume of 473 ml) to determine their fumigant toxicity. Thymol was the most toxic compound with a LC_50_ value of 20.50 mg/L (Table 2). Carvacrol (LC_50_ = 46.3 mg/L) and linalool (LC_50_ = 51.2 mg/L) were less toxic than thymol followed by (±)-camphor, menthone, eucalyptol, (−)-terpinen-4-ol, trans-cinnamaldehyde, R (+)-limonene, α-pinene, (±)-citronellal, geraniol, citronellic acid, eugenol and methyl eugenol based on the relative median potency analysis (Table S3). Dichlorvos (DDVP), an organophosphate insecticide with fumigant properties was used as a positive control. DDVP was 445 times more potent (LC_50_ value of 0.0432 mg/L) than thymol (Tables 2 and S3).

**Table 2:** The mortality response of adult male bed bugs exposed to vapors of essential oil components and dichlorvos and their corresponding percent evaporation values for the 24 h bioassay period.

Essential oil components	N	LC_50_^a^, mg/L (95% FL^b^)	Slope ± SE	χ²	Df	*P-*value	% Evaporation^c^
Thymol	180	20.50 (17.70–23.18)a	2.19 ± 0.29	0.60	3	0.89	90 ± 4.86
Carvacrol	180	46.3 (37.8–54.9)b	1.37 ± 0.15	8.65	5	0.124	26.89 ± 4.23
Linalool	240	51.2 (41.3–70.0)b	1.09 ± 0.19	6.33	3	0.097	86 ± 6.77
(±)-Camphor	210	133.3 (106.9–157)c	1.93 ± 0.28	5.86	4	0.209	52.99 ± 23.95
Menthone	270	150.7 (132.3–169.3)cd	2.03 ± 0.23	4.70	6	0.58	60.22 ± 20.33
Eucalyptol	180	191.1 (168.3–213.8)d	2.77 ± 0.37	11.62	3	0.009	100
(−)-Terpinen-4-ol	210	388.3 (301.7–482.9)e	0.96 ± 0.13	4.04	4	0.40	24.43 ± 16.55
trans-Cinnamaldehdye	240	389.0 (304.5–482.9)e	0.90 ± 0.11	8.82	5	0.116	0.50 ± 0.50
R (+)-Limonene	270	454.0 (436.5–476.5)e	6.69 ± 1.09	14.63	6	0.023	73.11 ± 9.25
α-Pinene	300	488.8 (470.8–503.6)e	8.45 ± 1.10	23.71	7	0.001	87.36 ± 7.27
(±)-Citronellal	180	1474.6 (1047.7–2528.1)f	0.63 ± 0.11	5.00	4	0.286	21.57 ± 12.03
Geraniol^†^	180	ND					1.29 ± 0.53
Citronellic acid^†^	180	ND					4.48 ± 1.22
Eugenol^†^	180	ND					5.16 ± 2.73
Methyl eugenol^†^	180	ND					0.65 ± 0.17
**Positive control**							
DDVP	270	0.0432 (0.0397–0.0468)g	2.76 ± 0.32	4.08	6	0.665	95.95 ± 4.04

^a^LC_50_ = median lethal concentration (expressed as amount of essential oil constituents or insecticides per liter air i.e. mg/L) necessary to kill 50% of individuals. ^b^95% FL = 95% fiducial limits. Daggers (^†^) show essential oil components for which accurate LC_50_ values were not determinable (ND) because less than 30% mortality was observed at the highest concentration (2000 mg/L) that was testable. LC_50_ values with the same letters are not significantly different based on the relative median potency analysis (refer Table S3 for details). Mortality in control groups was 0%. ^c^Percent evaporation values for the 24 h bioassay period. Acetone applied to control filter papers evaporated completely (100%) during the 30 sec to 5 min drying period described in the methods section.

Acetone (solvent carrier) applied to control filter papers evaporated completely (100%) during the 30 sec to 5 min drying time described in the methods section. Data on evaporation of different essential oil components for the 24 h bioassay duration are presented in Table 2. Percent evaporation was highest for eucalyptol (100%), whereas it varied from ~90% for thymol to <1% for trans-cinnamaldehyde. When regression analysis was performed between compounds for which LC_50_ values were accurately determinable, i.e., the first 11 compounds shown in Table 2 and their percent evaporation values no significant correlation was observed (P > 0.05; Fig. S1). Similarly, regression analysis between the four most toxic fumigant compounds and evaporation percentage did not reveal any significant correlation (P > 0.05; Fig. S1).

### Neurophysiology study

**Figure 1:**
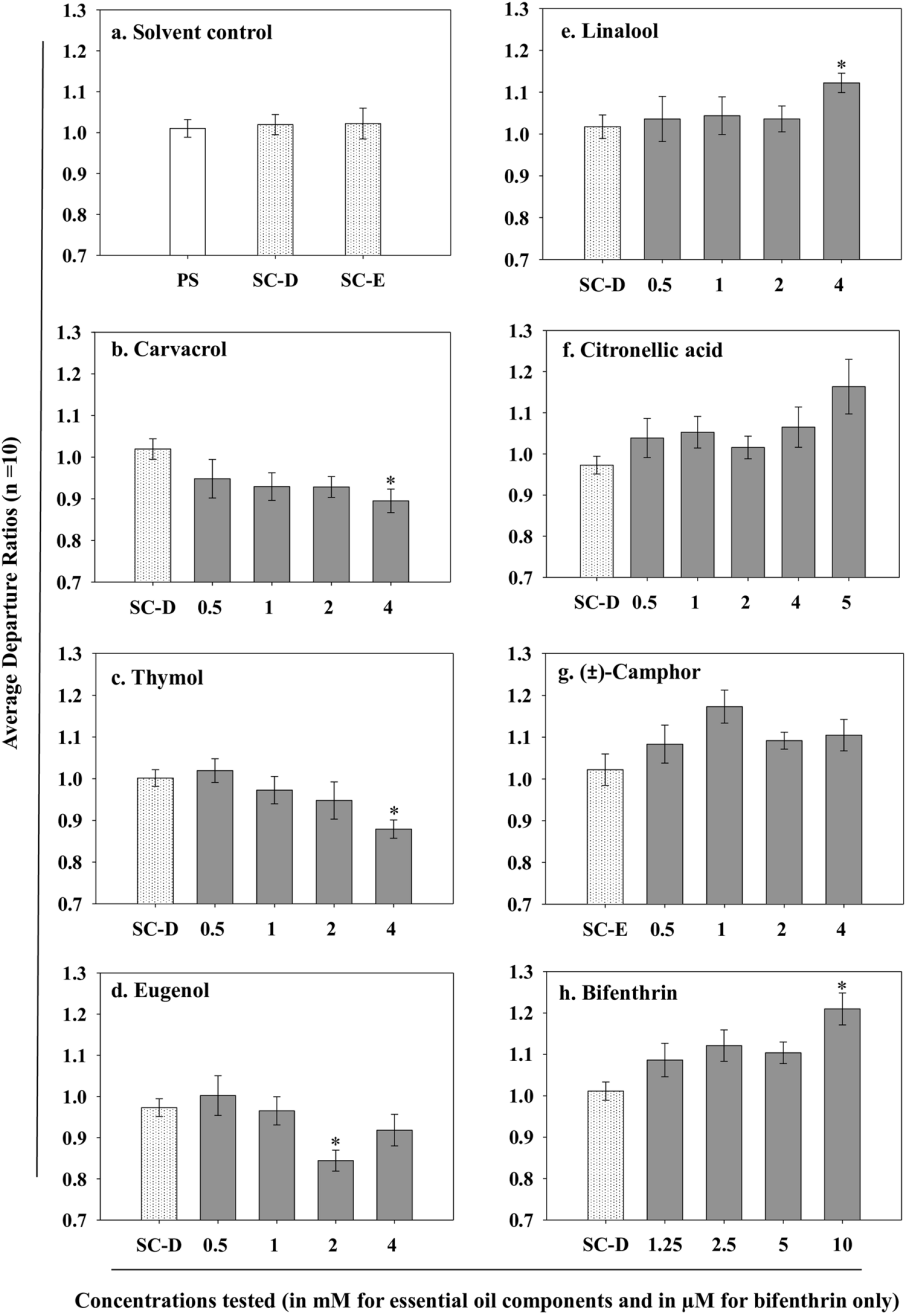
Neurophysiological effects of essential oil components, bifenthrin and solvent controls on the bed bug nervous system. Bars represent average departure ratios calculated by dividing the nervous activity spikes surpassing the threshold in post-treatment recordings (either with essential oil constituents or bifenthrin or solvent controls) with spike counts from physiological saline (PS) pre-treatment. Asterisks (*) in different graphs indicate significant differences from solvent control recordings (two-sample t-tests with Bonferroni corrected P-value i.e. 0.05 ÷ number of comparisons for each compound). (**a**) Solvent control treatments, PS + 0.1% dimethyl sulfoxide (DMSO) + 0.01% Tween-20 (SC-D) or PS + 0.1% absolute ethanol + 0.01% Tween 20 (SC-E) had no effect on nervous system activity (P > 0.025). (**b**) Carvacrol (4 mM), (**c**) thymol (4 mM), and (**d**) eugenol (2 mM) exhibited a neuroinhibitory effect as indicated by departure ratios significantly below 1 (P < 0.0125). (**e**) With departure ratios above 1, linalool (4 mM) led to significant neuroexcitation (P < 0.0125), but (**f**) citronellic acid (P > 0.01) and (**g**) (±)-camphor (P > 0.0125) did not cause significant neurological impacts. (**h**) The positive control treatment with bifenthrin (10 µM) caused significant neuroexcitation (P < 0.0125).

Spontaneous nerve activity recordings from the fused thoracic ganglion of adult male bed bugs demonstrated no neuroexcitatory or neuroinhibitory effects of solvent controls containing either 0.1% DMSO + 0.01% Tween 20 (P = 0.790) or 0.1% absolute ethanol + 0.01% Tween 20 (P = 0.826) in comparison to the HEPES-buffered physiological saline (PS) treatment (Fig. 1a). At the Bonferroni adjusted statistical significance level of P < 0.0125 (i.e., 0.05 ÷ number of comparisons in two-sample t-tests) the concentration of 4 mM for both carvacrol (P = 0.005) and thymol (P = 0.001) caused significant neuroinhibition (Fig. 1b,c). Eugenol exhibited significant neuroinhibitory effects at the 2 mM concentration (P = 0.001; Fig. 1d).

For linalool, the concentration of 4 mM (P = 0.011) produced significant neuroexcitatory effects (P < 0.0125) (Fig. 1e). Citronellic acid (Fig. 1f) and (±)-Camphor (Fig. 1g) resulted in departure ratios that were >1 and were indicative of neuroexcitatory effects, however, none of the concentrations tested for these compounds caused a significant increase in nervous activity at the Bonferroni corrected significance levels of P < 0.01 and P < 0.0125, respectively. As expected, the positive control treatment with bifenthrin (a synthetic pyrethroid insecticide), caused significant neuroexcitation at the 10 µM concentration (P = 0.0001; Fig. 1h).

Linear regression analysis showed that carvacrol and thymol caused a concentration-dependent decrease in spontaneous electrical activity of the nervous system (P < 0.05, Fig. S2). In contrast, citronellic acid, linalool and bifenthrin induced concentration-dependent increase in nervous activity (P < 0.05, Fig. S2). Eugenol and (±)-camphor did not show concentration-dependent changes in neurological activity (P > 0.05, Fig. S2), likely because their effects were bi-phasic (i.e., pronounced effects at intermediate concentrations in comparison to lower or higher concentrations; Fig. 1d,g).

### Poisoning symptoms (non-quantitative) in bed bugs treated with plant essential oil components

Treatment of bed bugs with the solvent carrier (acetone) did not induce any poisoning symptoms such as hyperactivity, paralysis or leg tremors at 2 and 4 h after treatment (Table 3). However, hyperactivity, defined as uncoordinated movement and wandering behavior, was observed in bed bugs treated with five of the six most toxic essential oil components (carvacrol, thymol, eugenol, linalool and (±)-camphor) at the 2 h interval (Table 3). Citronellic acid treated insects did not show hyperactivity symptoms. Bed bugs treated with all six toxic plant essential oil components were paralyzed, i.e., they were unable to walk or right themselves upon prodding at the 4 h observation interval (Table 3). Paralysis was also observed in thymol and (±)-camphor treated insects 2 h after treatment. Leg tremors (involuntary leg spasms, twitching and quivering) were observed in knocked-down insects treated with thymol, linalool and (±)-camphor (Table 3). Death of treated insects was first observed ~6 h after treatment with some of the compounds and hence observations on non-quantitative poisoning symptoms were not recorded after the 4 h observation interval.

**Table 3:** Poisoning symptoms observed in bed bugs topically treated with median lethal dose (LD_50_) of six most toxic plant essential oil components.

Essential oil components	Hours after treatment	Poisoning symptoms (0 = absent, + = present)		
		Hyperactivity	Paralysis	Leg tremor
Control (acetone)	2	0	0	0
	4	0	0	0
Carvacrol	2	+	0	0
	4	+	+	0
Thymol	2	+	+	+
	4	0	+	+
Eugenol	2	+	0	0
	4	0	+	0
Citronellic acid	2	0	0	0
	4	0	+	0
Linalool	2	+	0	0
	4	0	+	+
(±)-Camphor	2	+	+	+
	4	0	+	+

Acetone treated insects did not display poisoning symptoms. However, poisoning symptoms such as “hyperactivity” (insects displaying uncoordinated movement and wandering behavior), “paralysis” (insects that were either unable to walk or knockdown insects that were unable to right themselves upon prodding) and “leg tremors” (insects lying on their back and exhibiting involuntary leg spasms, twitching and quivering), were observed in bed bugs treated with essential oil components.

## Discussion

Initially we characterized the inherent toxicity of fifteen different plant essential oil components against bed bugs. Carvacrol and thymol were the most active compounds in topical application bioassays. Both compounds exhibited similar levels of contact toxicity and were 13–15 times more potent than the least toxic constituent, methyl eugenol in topical bioassays. Carvacrol and thymol were previously reported as being effective, with contact and fumigant toxicity against several insect pests including cockroaches, kissing bugs and house flies^22–26^. As found in other insects, increased toxicity of carvacrol and thymol towards bed bugs might be due to two major properties: (i) they are saturated compounds (contain carbon-carbon single bonds outside the benzene ring) and (ii) the presence of functional hydroxyl groups on the benzene ring^22,25^. These structural properties may also have allowed thymol and carvacrol to penetrate rapidly through the cuticle, undergo slow detoxification and interact effectively with their target sites^22,25,40^. The lipophilicity of essential oil compounds is another important property that plays a role in penetration through the insect cuticle^22^. The LogP or octanol-water partition coefficients (higher values indicate greater lipophilicity)^25^ for carvacrol were higher than thymol (Table S1). Similarly, the LogP coefficient for the third most toxic compound in topical assays (citronellic acid) was higher than the LogP coefficient for eugenol (Table S1). In previous studies, citronellic acid and eugenol have been shown to possess contact toxicity against *Musca* *domestica* and *Tetranychus urticae*^22,28^.

When considering the fumigant toxicity of essential oil constituents, thymol was most potent, followed by carvacrol, linalool, and (±)-camphor (Table 2). As stated in the previous paragraph, thymol and carvacrol have contact and fumigant toxicity against several insect species^22–26^. Fumigant effects of linalool have been demonstrated against *Thrips palmi*, *Plutella xylostella* and *Blattella* *germanica*^27,29,30^. Whereas, (±)-camphor was reported as having contact and fumigant action against the *P*. *xylostella*^30^, but not against stored product pests^31^.

Determination of 24 h evaporation levels for essential oil constituents revealed large variations among compounds. The amount of initially applied chemical that evaporated during the 24 h bioassay period ranged from <0.5% for trans-cinnamaldehyde to 100% for eucalyptol (Table 2). A series of regression analyses conducted between LC_50_ values and evaporation percentage showed no significant correlations (P > 0.05; Fig. S1). Interestingly, the constituents for which LC_50_ values were not determinable (geraniol, citronellic acid, eugenol and methyl eugenol) (Table 2) generally showed <5% evaporation during the 24 h bioassay period. However, for trans-cinnamaldehyde, which showed lowest evaporation percentage of 0.5%, a LC_50_ value was still determinable (Table 2). In the case of carvacrol, its evaporation estimate for the bioassay period was ~27%, but it was the second most toxic fumigant. In contrast, eucalyptol completely evaporated in 24 h, but was the sixth most toxic compound. Collectively, these results indicated that the fumigant toxicity of the tested essential oil components was not solely dependent on their volatility, but their inherent toxicity (i.e., unique target-receptor interactions) was likely a major determining factor in their toxicity. Since fumigant toxicity is dependent on the exposure time, in the future it may be important to perform experiments to determine if compounds with low evaporation show increased toxicity against bed bugs in long duration bioassays (3–7 d) as shown by Feldlaufer and Ulrich^19^ when using pure essential oils.

Several essential oil-based products have already been commercialized, especially for bed bug control. However, of the nine different natural compound products, only EcoRaider^®^ (active ingredients: geraniol (1%), cedar extract (1%), and sodium lauryl sulfate (2%)) and Bed Bug Patrol^®^ (active ingredients: clove oil (0.003%), peppermint oil (1%), and sodium lauryl sulfate (1.3%)) were reported to be effective in laboratory and field experiments conducted against bed bugs^11,21^. Carvacrol and thymol were the most active compounds in our assays but are not present in any of the essential oil-based products available for bed bug control. Therefore, based on the findings of this study there are opportunities to develop potentially efficacious essential oil-based formulations for use in bed bug IPM. Plant essential oils that contain high concentrations of effective compounds included in this study were found active against bed bugs and cockroaches^19–21,26^. Therefore, thyme (*Thymus vulgaris* L.) and oregano (*Origanum vulgare* L.) plant essential oils, which contain high amounts of thymol, and carvacrol, respectively (Table S1) can be included in the formulation of natural product insecticides. The odor issue associated with the use of essential oils in indoor environments can be alleviated by formulating with inert carriers, surfactants, adjuvants and additives. Most prior work with commercial essential oil products has been performed by conducting direct spray and residual exposure bioassays, but no study has evaluated fumigant activity under field conditions. Thymol was more potent as a fumigant than any other essential oil constituent tested in this study. Therefore, thymol or thymol containing essential oils have the potential of being used as fumigants for bed bug control under field settings. For example, small bed bug infested items can be sealed in chambers or plastic bags with a paper or cloth impregnated with essential oils containing thymol^19^.

Electrophysiology recordings were performed using the suction electrode technique to investigate the effects of essential oil components on the bed bug nervous system. Four of the six most active components identified collectively from topical and fumigant bioassays impacted baseline electrical activity of the bed bug nervous system. The neurophysiology data for carvacrol, thymol, eugenol and linalool provides a basis for understanding their toxicity against bed bugs. Bifenthrin (a positive control insecticide used in this study) and other synthetic pyrethroids modify the gating characteristics of voltage-sensitive sodium channels that lead to a delay in their closure, and thereby cause a neuroexcitatory effect on the insect nervous system^41^. In this study, bifenthrin caused significant neuroexcitation of baseline nervous system activity. Effects of bifenthrin at the 10 µM concentration on the bed bug nervous system were similar with a study that employed the suction electrode electrophysiology technique against the mole crickets^42^. Both neuroexcitatory (linalool) and neuroinhibitory (carvacrol, thymol and eugenol) essential oil constituents were neurologically active at millimolar (mM) concentrations. The structural and chemical property differences between essential oil components and bifenthrin may have led to significant differences in toxicity at the nervous system level^40^. In this regard, higher lipophilicity of bifenthrin (LogP value of 6, Table S1) in comparison to that of essential oil constituents may allow bifenthrin to effectively penetrate and interact with the membrane bound target site(s) within the nervous system at micromolar concentrations. Overall, low potency of neurological effects caused by essential oil compounds is consistent with their relatively lower topical and fumigant toxicity to different insect pest species and bed bugs. The effective concentration range or quantity of essential oil components (2 to 4 mM or 1.5 × 10^−11^ to 3.4 × 10^−10^ µg/insect or nerve preparation) necessary to produce statistically significant neurological effects was at least 1 billion times lower in comparison the topical LD_50_ estimates that ranged from 54–1120 µg/insect or 27–560 µg/mg body weight (Table 1). Large differences in effective quantities or doses of essential oil components between neurophysiology and whole organism bioassays were expected. This is because toxicants that are directly applied to nerves do not have to penetrate the cuticle, and thereby have less likelihood of being degraded or sequestered by detoxification enzymes before reaching their target site^40^. In bed bugs, detoxification enzymes expressed in the cuticle have been associated with rapid degradation of insecticides^4^. Therefore, different insecticides, including essential oil components are effective at lower concentrations when directly applied to the ventral nerve cord.

Neurological impacts of essential oil components against bed bugs were concentration-dependent for most test compounds (P < 0.05; Fig. S2). Similarly, Price and Berry^38^ found concentration-dependent neurological effects of essential oil components on the ventral nerve cord of *P*. *americana* and *B*. *discoidalis*. The effective concentration ranges for essential oil constituents tested in this study were similar to those of Price and Berry for citral, eugenol and geraniol^38^. The neurological impacts of eugenol and (±)-camphor were not concentration-dependent and showed a biphasic effect in our study (Figs 1 and S2). A previous study also revealed biphasic effects of geraniol on cockroach nervous system activity^38^.

The three compounds that produced neuroinhibition were carvacrol, thymol and eugenol. Based on *in vitro* studies, carvacrol is known to inhibit *M*. *domestica* nAChRs^37^ and its inhibitory activity was similar to dinotefuran (a neonicotinoid insecticide)^43^. In vertebrates, carvacrol can reversibly block the excitability of the rat sciatic nerve in a dose-dependent pattern^44^. However, in previous studies with insects, tyramine receptor^36^, transient receptor potential-like (TRPL) channels^45^ and GABA^46^ were also proposed as potential target sites for carvacrol. Thymol has been shown to bind *Drosophila* *melanogaster*, mouse and human GABA receptors^35,47,48^. It was also reported as a weak inhibitor of the acetylcholinesterase enzyme^49,50^. Eugenol, which is a phenolic compound, was previously reported to have neuroinhibitory effects on *P*. *americana* and *B*. *discoidalis*^38^ and it was proposed to bind or interact with octopamine receptors in the insect nervous system^34,51^.

Linalool produced neuroexcitatory effects on the bed bug nervous system (Fig. 1e). Linalool was initially reported to act as a reversible competitive inhibitor of the acetylcholinesterase enzyme^52^. However, in subsequent studies, it was concluded that linalool does not bind to neurotransmitter enzymes^53,54^. It also did not produce any effect on house fly [^3^H]-TBOB ([3 H]-t-butylbicycloorthobenzoate) binding and *P*. *americana* ^36^Cl^−^ uptake studies^46^. Although, citronellic acid caused a concentration-dependent increase in nervous activity (P < 0.05; Fig. S2) and resulted in a 6–19% increase in activity of the nervous system, two-sample t-tests with Bonferroni adjustment revealed that none of the tested concentrations caused a statistically significant increase in nervous activity (P > 0.01). Thus far, no target site or neurological impact data are available for citronellic acid. Lastly, camphor has been shown to inhibit catecholamine secretion by blocking nACHRs in bovine adrenal chromaffin cells^55^. In another study with stored product pests and *B*. *germanica*, camphor was a weak acetylcholinesterase inhibitor^54,56^. However, in this study the 6–15% increase in nervous activity induced by (±)-camphor at various concentrations was not statistically significant (Fig. 1g). Given these findings for citronellic acid and (±)-camphor, more sensitive electrophysiology techniques such as patch or two-electrode voltage clamping may be required to determine the actual neurological impacts of these constituents. Target site binding studies may also help in determining the neurotoxic nature of these compounds.

Bed bugs treated with the six most toxic plant essential oil components showed a range of poisoning symptoms such as hyperactivity, paralysis and leg tremors. Previously, Coats *et al*.^57^ reported hyperactivity and leg tremors as common poisoning symptoms associated with essential oil constituents. In the Madagascar cockroach (*Gromphadorhina portentosa*), pulegone-1,2-epoxide (an essential oil component) caused hyperactivity and muscular spasms before eventual paralysis and death^58^. In general, neuroinhibitory insecticides (e.g., oxadiazines and avermectins) are known to cause flaccid paralysis, wherein the muscles become limp and are unable to contract due to reduction or loss of nerve activity^40,59^. In contrast, rigid paralysis is caused by neuroexcitatory insecticides (e.g. organophosphates, pyrethroids, neonicotinoids)^40,59^. Rigid paralysis occurs because of the overstimulation of nervous system activity that causes muscles to stay in a contracted state. However, such symptoms were not visually distinguishable in bed bugs treated with neuroinhibitory (carvacrol, thymol and eugenol) or neuroexcitatory (linalool) essential oil components.

In summary, baseline toxicity of essential oil components against bed bugs as reported here provides information for development of natural product insecticides that can be used in bed bug IPM. Electrophysiology data for the most active compounds from bioassays further verifies that certain essential oil constituents affect the normal functioning of the bed bug nervous system. Collectively, these results provide insights required for identifying the target or binding sites and mode-of-action of specific essential oil constituents.

## Materials and Methods

### Insects

**Figure 2:**
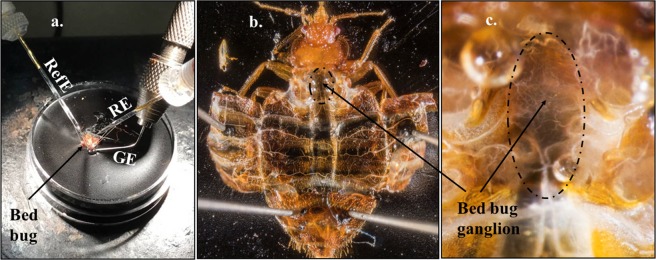
Electrophysiology recording set-up (suction electrode technique), dissected bed bug and its ganglion. (**a**) Recording electrode (RE) was placed in gentle contact with the fused thoracic ganglion, whereas the reference electrode (RefE) was placed in contact with the carcass. The ground electrode (GE) was placed in the Petri dish, but in contact with the external cuticle of the bed bug body in the presence of saline. (**b**,**c**) Fused thoracic and abdominal ganglion of the bed bug can be seen in the metathoracic region. Segmental nerves extend from the fused ganglion (*see* reference number 61 for a description of the bed bug ventral nerve cord).

The susceptible Harold Harlan strain of bed bug was used for all experiments. This strain was maintained at 25 °C, 50 ± 15% relative humidity, and a photoperiod of 12:12 (L: D) h. Bed bugs were fed weekly on defibrinated rabbit blood (Hemostat Laboratories, Dixon, CA) using the membrane feeding method^60^. Each week, 5^th^ instar nymphs were separated from the main colony and reared in different jars. Newly emerged adult males were separated and used in all experiments. For toxicity evaluation, 8–10 d old adult males were used (average weight = ~2 mg per insect) that were fed 4–5 d before bioassays. However, for electrophysiology studies 10–15 d old adult males that were fed 7–8 d before evaluation were used. This starvation period allowed for clean dissections due to the absence of undigested blood in the foregut and midgut (Fig. 2b).

### Chemicals

High purity essential oil components carvacrol, geraniol, eugenol, methyl eugenol, trans-cinnamaldehyde, citronellic acid, (±)-citronellal, α-pinene, linalool, R (+)-limonene, eucalyptol, (−)-terpinen-4-ol, and menthone were obtained from Sigma-Aldrich (St. Louis, MO), whereas thymol and (±)-camphor were obtained from Alfa Aesar (Hill, MA) (Table S1). These active constituents are found in various aromatic plants (Table S1). All fifteen essential oil components (Table S1) were selected based upon the previous toxicity literature on different urban and agricultural pests^22–31^. The positive controls dichlorvos (≤100% purity) and bifenthrin (98% purity) were obtained from Sigma Aldrich and Chem Service Inc. (West Chester, PA), respectively. Analytical grade solvents such as acetone, ethanol and dimethyl sulfoxide (DMSO) were purchased from Fisher Scientific (Pittsburgh, PA). Buffer salts and other reagents used for preparation of HEPES (4-(2-hydroxyethyl)−1-piperazineethanesulfonic acid)-buffered physiological saline were purchased from Sigma-Aldrich, Fisher Scientific and Avantor Performance Materials, LLC (Center Valley, PA).

### Topical application

Initially, each essential oil component was diluted in acetone on a volume-to-volume basis to prepare stock solutions based on the density of each component (Table S1). The only exceptions were thymol and (±)-camphor, which were prepared on a weight per volume basis due to their crystalline nature or form. The stock solutions were then serially diluted to prepare a range of dilutions (at least 5 for each component). Topical applications of different concentrations (volume range 0.5–1 µL) were made on the ventral metathorax using a 25 µL micro-syringe (Hamilton, Reno, NV) attached to a PB-600-1 repeating dispenser (Hamilton, Reno, NV). Insects were immobilized by attaching them dorsally to colored labelling tape (Fisher Scientific, Pittsburg, PA). Control groups were treated with acetone only. Technical grade bifenthrin dissolved in acetone (weight to volume basis) was used as a positive control. After treatment, insects (in groups of 10) were transferred into 35 × 10 mm Petri dishes with vents (Item number: 627161, Greiner Bio-One, Frickenhausen, Germany) lined with a single layer of Whatman # 1 filter paper (GE Healthcare UK Limited, Amersham Place, UK). Petri dishes were then placed in an environmental chamber with temperature, humidity and lighting conditions similar to those used for rearing. Initial bioassay experiments suggested that mortality caused by essential oil treatments did not significantly change between observation intervals of 24 and 48 h. Therefore, mortality scoring of all treatments was performed at 24 h post-treatment. Insects that were lying on their backs and/or were unable to move upon prodding were scored as dead. In total, three replicates were performed for each concentration (n = 30). The average weight of a single adult male bed bug used for bioassays was 2 mg. Hence, the topical lethal dose values are reported as µg/mg body weight.

### Fumigant exposure and quantification of evaporation for essential oil components

Filter papers (9 cm diameter, Whatman #1) (GE Healthcare UK Limited) were treated with essential oil component solution (volume range: 9.46–1892 µL) prepared in acetone as described under “Topical application” bioassays. Treated papers were placed in glass containers (473 mL Mason jars; Anchor Glass Container Corporation, Tampa, FL) after complete evaporation of acetone. Evaporation time varied from ~30 sec to 5 mins based upon insecticide volume that was applied to the filter paper. In case of dichlorvos, only 30–45 sec of evaporation time was required because the treatment volume of ~10–15 µL was much lower in comparison to that of essential oil components. Ten adult bed bugs held in a mesh-covered glass scintillation vial (20 mL; W.W. Grainger, Inc., Lake Forest, IL) were then placed in mason jars along with treated filter papers. The mason jar was then sealed completely and transferred to an environmental chamber. Control insects were exposed to acetone treated filter papers. Acetone application volume for controls corresponded to the volume used for highest insecticide concentration or application volume of each tested compound. Three replicates (n = 30) were performed for each concentration. Mortality did not significantly change after the initial 24 h observation interval, as such all observations were recorded 24 h post exposure. Mortality was scored by following the same protocol described for topical bioassays. Fumigant lethal concentration values are expressed as amount of insecticide per liter air volume (mg/L).

To determine essential oil constituent or DDVP evaporation levels during the 24 h bioassay period, we first measured the weight (in grams) of untreated filter papers (W0) on a Mettler AE 100 weighing scale (Mettler-Toledo, Inc., Columbus, OH). After that, acetone-diluted essential oil constituents or insecticides were applied to the filter paper and the weights of these treated filter papers were recorded after the acetone (solvent carrier) evaporation period (30 sec to 5 mins) described in the previous paragraph had elapsed (W1). Control filter papers were treated with acetone only. Filter papers were then placed individually in sealed mason jars for 24 h. At 24 h, filter papers were weighed again (W2). Three concentrations (low, medium and high) were used for determining evaporation percentage for each compound. They were representative of the entire range of concentrations tested in fumigant bioassays for each compound. Three independent replicates were performed for each concentration. The following formula was used for calculating percent evaporation:

$$ \% \,{\rm{Evaporation}}=\frac{{\rm{Amount}}\,{\rm{evaporated}}\,(\mathrm{W1}-\mathrm{W2})}{{\rm{Amount}}\,{\rm{applied}}\,(\mathrm{W1}-\mathrm{W0})}\times 100$$

### Electrophysiology equipment

The electrophysiology equipment used in this study was previously described by Gondhalekar and Scharf ^61^ and Feston^62^. The setup consists of three electrodes; recording, reference and ground (Fig. 2a). Recording and reference electrodes were mounted on suction electrode holders (Cat. No. 64–1035 Warner Instruments, Hamden, CT). Both electrodes were fabricated from ~4 cm lengths of 0.5 mm diameter gold wire (World Precision Instruments, Sarasota, FL) and fitted within 1.0 mm borosilicate glass capillaries (Harvard Apparatus, Holliston, MA) that were pulled to a fine point with a Micropipette puller (Narishige Co., LTD, Tokyo, Japan). Capillaries were used only for single recordings. The ground electrode consisted of #2 steel pin (Catalog #1208B2 Bio Quip Products, Rancho Dominguez, CA) which was held by a Pin Vise (#162 A The L.S. Starrett Company Athol, MA). All electrodes were connected to a model 4001 capacitance compensation head stage (Dagan Inc., Minneapolis, MN), which was connected to a Hum Bug 50/60 Hz Noise Eliminator (Quest Scientific Instruments Inc., North Vancouver, BC, Canada) and then a model EX-1 differential amplifier (Dagan Inc., Minneapolis, MN). The amplifier was interfaced with computerized digitizing hardware (PowerLab/ 4SP, ADInstruments, Milford, MA) and software that functioned as an eight-channel chart recorder (Chart version 3.5.7, ADInstruments, Milford, MA).

### Dissections and neurophysiology recordings

Dissections were performed in 35 × 15 mm Petri dishes (Fisher Scientific, Hampton, NH) filled 2/3 of their volume with wax (Frey Scientific and CPO Science, Nashua, NH) (Fig. 2a) under a Leica S6D Greenough stereo microscope (Leica Microsystems Inc. Buffalo Grove, IL). Bed bugs were immobilized by four 0.15 mm stainless minutien pins (Carolina Biological Supply Company, Burlington, NC) during dissection (Fig. 2b). New Petri dishes and minutien pins were used for each recording. The general procedure described by Feston^62^ was used for performing dissections. Each experimental bed bug was dissected via one longitudinal incision from the dorsal abdomen up to the thorax followed by two latitudinal incisions across the wing pads to expose the fused ganglion (Fig. 2b)^63^. One microliter of HEPES-buffered saline, pH 7.1 was pipetted into the insect hemocoel immediately after dissection. Fat bodies, gut and other thoracic and abdominal body tissues were removed for better visualization of the ganglion (Fig. 2b,c).

**Figure 3:**
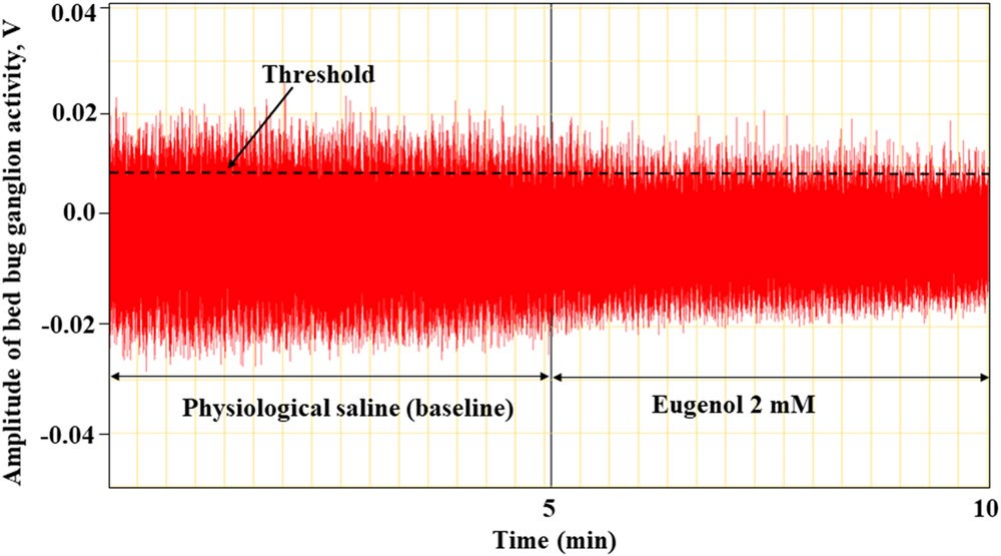
An example of 10-minute electrophysiological nerve activity trace from the Chart Software for 2 mM eugenol. Determination of spontaneous electrical activity bursts or spikes in pre-treatment or baseline recordings in physiological saline (for 5 mins) and post-treatment recordings in 2 mM Eugenol (5 mins) were enabled by setting the threshold using the “counter” function in the Chart software. The threshold was maintained constant between the pre-and post-treatment recordings. Data for the total number of spikes surpassing the threshold before and after treatment were used to calculate ratios representing a departure from baseline activity.

Baseline electrical or nerve activity recordings were performed in HEPES-buffered physiological saline (volume: 1.5–2 µL; 185 mM sodium chloride, 10 mM potassium chloride, 5 mM HEPES sodium salt, 5 mM calcium chloride, 5 mM magnesium chloride and 20 mM glucose; pH 7.1)^61,62,64^. The recording electrode, fitted with a pulled glass capillary and filled with HEPES-buffered saline, was placed in gentle contact with the fused ganglia (Fig. 2a–c) with the help of a micromanipulator (model MNJR, World Precision Instruments). The reference electrode was prepared identically and placed in contact with the carcass (Fig. 2a). A ground electrode was placed in the dissection dish outside the bed bug carcass, but in contact with physiological saline (Fig. 2a). The total electrical activity recording for each insect was done for 10 minutes (Fig. 3). For the first 5 mins, spontaneous pretreatment electrical activity (*i*.*e*., baseline) was recorded by setting a threshold for the “counter” function on the Chart software (Fig. 3). The baseline electrical activity recording in physiological saline was briefly paused after the first 5 mins to enable application of 1 µL of essential oil component solution gently onto the ganglion. Multiple concentrations of essential oil constituents ranging from 0.5 to 5 mM were tested (approx. 0.5 to 5 mM or 3.75 × 10^−12^ to 4.25 × 10^−10^ µg of constituent per insect preparation). This solution was prepared by diluting essential oil components initially in DMSO (used for thymol, carvacrol, eugenol, citronellic acid and linalool dilution) or ethanol (used for (±)-camphor dilution) and then further dilutions were made in physiological saline containing 0.01% Tween 20. Recordings were resumed approximately 10–15 sec after the application of essential oil-containing solution. The waiting period of 10–15 sec was included to allow the ganglion to recover from the physical disturbance (if any) caused by application of 1 µL essential oil constituent solution. The threshold for the “counter” function remained constant for the 5 min pre-treatment and 5 min post-treatment nerve activity recordings (Fig. 3). For control recordings, a solution containing physiological saline + 0.1% DMSO or 0.1% ethanol + 0.01% Tween 20, but no essential oil component was used. To compare or see the effect of solvent controls on nervous activity, recordings were performed using physiological saline for the 5 min pre-treatment and 5 mins post-treatment recordings.

Departure ratios that represent deviation from the baseline electrical activity were calculated by dividing the total number of spike counts surpassing the threshold in post-treatment 5 min recordings (with essential oil constituents) with the total number of spike counts above threshold in 5 min of pre-treatment recordings (with physiological saline). Departure ratios that were significantly greater than “1.0” indicated neuroexcitatory action and ratios that were significantly less than “1.0” were indicative of neuroinhibition^61^. Similar procedures were followed to calculate departure ratios for solvent control preparations.

For the positive control treatment using bifenthrin (a pyrethroid insecticide), the same procedures were followed, however, the treatment volume was higher (2 µL). The use of a higher volume was necessary for bifenthrin based on preliminary experiments. In a preliminary study, 1 µL volume of 1.25–10 µM bifenthrin did not significantly excite the bed bug ventral nerve cord. Each bed bug or dissection represented one replicate and ten replications were performed for each essential oil component or positive control (bifenthrin) concentration, solvent controls and physiological saline controls. The recordings in which bed bugs were dead during or after 10 minutes were discarded and a new recording was performed with a new insect preparation to account for the loss.

### Topical bioassays to observe poisoning symptoms

To observe poisoning symptoms at the whole organism level caused by the six most toxic essential oil components, topical application bioassays were performed at the LD_50_ for each compound. Acetone-diluted compounds were applied to the metathoracic region using identical procedures outlined for “Topical application” bioassays. Control insects were treated with acetone. Poisoning symptoms exhibited by adult male bed bugs were observed at 2 and 4 h post treatment either directly in the bioassay Petri dish or under a microscope. Short videos (~30 secs) of bed bugs from various treatments were also recorded at the 2 and 4 h intervals and were used to confirm or cross-check the presence or absence poisoning symptoms. In total 30 insects were observed for each compound. Specifically, the presence or absence of three symptoms was observed: (1) hyperactivity (uncoordinated movement and wandering behavior), (2) paralysis (inability to walk or right themselves up on prodding) and (3) tremors (insects lying on their back and exhibiting involuntary leg spasms, twitching and quivering).

### Statistical analysis

Probit analysis was performed on dose-mortality and concentration-mortality data from topical application and fumigant exposure bioassays to calculate LD_50_ and LC_50_ values, respectively and their 95% fiducial limit (FL)^65^. Relative median potency analysis was performed to statistically compare toxicity differences between the compounds^66,67^. The LD_50_ or LC_50_ values between different compounds are significantly different (P < 0.05) if confidence intervals (CIs) for toxicity ratios did not overlap with 1^66,67^. For the electrophysiology study, departure ratios calculated for essential oil components or bifenthrin were log transformed after adding the value one (1) to all departure ratios. The addition of “1” to all departure ratio values allowed us to obtain positive log transformed data, i.e., to prevent negative log transformed values. Log transformed departure ratio data for different compounds were analyzed using linear regression to determine if they caused concentration-dependent increases or decreases in nervous system activity (P < 0.05). Departure ratios determined for solvent controls (DMSO and ethanol) were statistically compared to the physiological saline treatment using two-sample t-tests with a Bonferroni adjusted significance level of P < 0.025 (0.05 ÷ number of comparisons or tests)^68,69^. Bonferroni corrected two-sample t-tests were also used for conducting pairwise comparisons between log transformed departure ratio data for solvent controls and various concentrations tested for essential oil compounds or bifenthrin^68,69^. Relative median potency analysis of topical and fumigant toxicity was performed using SPSS Version 25. All other statistical analysis, including LD_50_ and LC_50_ estimation was done using Minitab Software Release 14.2 (Minitab Inc. State College, PA).

## Supplementary information

Supplementary Information File
