# Supplementary material for: Toxicity and neurophysiological impacts of plant essential oil components on bed bugs (Cimicidae: Hemiptera)
Source: Sci Rep. 2019 Mar 8;9:3961. doi: 10.1038/s41598-019-40275-5 (PMC6408565; doi:10.1038/s41598-019-40275-5)
Supplement: Supplementary file 1 — Supplementary Information File [file 41598_2019_40275_MOESM1_ESM.pdf]

Supplementary Information of

Toxicity and neurophysiological impacts of plant essential oil components on bed bugs (Cimicidae: Hemiptera)

Sudip Gaire, Michael E. Scharf and Ameya D. Gondhalekar*

Department of Entomology, Purdue University, West Lafayette, IN, 47907, USA
*Corresponding Author: ameyag@purdue.edu

Functional group	Essential oil components	Molecular formula[a]	Purity (%)	Molecular weight[a]	Density (g/ml) at 25°C[a]	Boiling point (°C)[a]	Vapor pressure at 25°C (mmHg)[a]	Partition Coefficient (LogP)	Plant sources with high proportion of respective compound[c]
Phenol	Carvacrol	$C_{10}H_{14}O$	≥ 98	150.22	0.976	236	0.0296	3.49[b]	Red thyme oil (*Thymus vulgaris* L.)/ Oregano oil (*Origanum vulgare* L.)
	Thymol	$C_{10}H_{14}O$	≥ 99	150.22	0.965	232	0.016	3.3	Red thyme/ oregano oil
	Eugenol	$C_{10}H_{12}O_2$	99	164.20	1.067	254	0.0221	2.7	Clove bud oil (*Syzygium aromaticum* L.)
Acid	Citronellic acid	$C_{10}H_{18}O_2$	98	170.25	0.923	121-122	0.005	3[b]*	Citronella oil (*Cymbopogon winterianus* Jowitt)
Alcohol	Geraniol	$C_{10}H_{18}O$	98	154.25	0.879	229-230	0.03	2.5	Rose (*Rosa × damascena* Mill.) /citronella oil
	Linalool	$C_{10}H_{18}O$	97	154.25	0.87	194-197	0.17	2.84	Basil oil (*Ocimum basilicum* L.)
	(–)-Terpinen-4-ol	$C_{10}H_{18}O$	≥ 95	154.25	0.934	209[b]	0.04[b]	3.26	Tea tree oil (*Melaleuca alternifolia*)

Hydrocarbon	α-Pinene	$C_{10}H_{16}$	98	136.23	0.858	155-156	4.75	4.48	Pine tree oil (*Pinus cembra* L.)
	R (+)-Limonene	$C_{10}H_{16}$	97	136.23	0.844[b]	176-177	1.98[b]	4.2	Citrus oil (*Citrus nobilis*)
Ketone	Menthone	$C_{10}H_{18}O$	97	154.25	0.896	85-88	0.28[b]	3.05	Peppermint oil (*Mentha piperita* L.)
	(±)-Camphor	$C_{10}H_{16}O$	96	152.24	0.992	204	0.65	2.38[b]	Camphor oil (*Cinnamomum camphora* L.)
Ether	Eucalyptol	$C_{10}H_{18}O$	99	154.25	0.921	176-177	1.9	2.74[b]	Eucalyptus oil (*Alpinia kwangsiensis*)
Aldehyde	trans-Cinnamaldehyde	C_9H_8O	99	132.16	1.05	250-252	0.0289[b]	1.9	Cinnamon oil *(Cinnamomum zeylanicum* Blum)
	(±)-Citronellal	$C_{10}H_{18}O$	≥ 95	154.25	0.857	207	0.25	3.62	Citronella oil
Phenylpropanoid	Methyl eugenol	$C_{11}H_{14}O_2$	98	178.23	1.036	242-255	0.012	3.03	Basil oil
**Chemical class**	**Positive controls**								
Pyrethroids Type I	Bifenthrin	$C_{23}H_{22}ClF_3O_2$	98	422.87	1.212	453.2	< 1	6	
Organophosphate	Dichlorvos (DDVP)	$C_4H_7Cl_2O_4P$	≤100	220.98	1.41	234.1	0.0158	1.43	

Supplementary Table S1. Properties and plant sources of essential oil components used in the study. [a]Properties presented in chemical "Safety Data Sheet" by respective company. [b]PubChem Open Chemistry database (https://pubchem.ncbi.nlm.nih.gov/). [c]Dr. Duke's Phytochemical and Ethnobotanical Databases (https://phytochem.nal.usda.gov). *refers computational prediction

Baseline essential oil components or insecticides	Essential oil components or insecticides for comparison	Relative median potency ratios (I)	Confidence intervals (CIs)	
			Lower limit	Upper limit
Carvacrol	Thymol	0.912	0.651	1.27
	Citronellic acid*	0.535	0.373	0.749
	Eugenol*	0.48	0.333	0.672
	Geraniol*	0.446	0.297	0.643
	α-Pinene*	0.351	0.229	0.512
	R (+)-Limonene*	0.284	0.18	0.422
	Linalool*	0.228	0.14	0.347
	Eucalyptol*	0.211	0.128	0.323
	(–)-Terpinen-4-ol*	0.203	0.118	0.323
	trans-Cinnamaldehyde*	0.173	0.103	0.27
	Menthone*	0.159	0.092	0.255
	(±)-Citronellal*	0.118	0.066	0.194
	(±)-Camphor*	0.044	0.02	0.083
	Methyl eugenol*	0.066	0.033	0.117
	Bifenthrin*	72076.002	8145.785	1058437.2
Thymol	Citronellic acid*	0.587	0.415	0.813
	Eugenol*	0.527	0.371	0.729
	Geraniol*	0.489	0.331	0.696
	α-Pinene*	0.385	0.257	0.553
	R (+)-Limonene*	0.311	0.202	0.456
	Linalool*	0.25	0.156	0.374
	Eucalyptol*	0.231	0.144	0.348
	(–)-Terpinen-4-ol*	0.222	0.133	0.348
	trans-Cinnamaldehyde*	0.19	0.116	0.291
	Menthone*	0.175	0.103	0.275
	(±)-Citronellal*	0.13	0.074	0.209
	(±)-Camphor*	0.048	0.023	0.089
	Methyl eugenol*	0.072	0.037	0.125
	Bifenthrin*	79060.069	8753.877	1191005.6
Citronellic acid	Eugenol	0.897	0.658	1.217
	Geraniol	0.832	0.594	1.149
	α-Pinene*	0.655	0.464	0.907
	R (+)-Limonene*	0.529	0.368	0.74
	Linalool*	0.425	0.289	0.601

	Eucalyptol*	0.393	0.265	0.56
	(–)-Terpinen-4-ol*	0.379	0.245	0.559
	trans-Cinnamaldehyde*	0.324	0.215	0.462
	Menthone*	0.298	0.191	0.439
	(±)-Citronellal*	0.221	0.138	0.331
	(±)-Camphor*	0.081	0.042	0.141
	Methyl eugenol*	0.123	0.07	0.198
	Bifenthrin*	134603.46	13625.857	2266382.6
Eugenol	Geraniol	0.928	0.669	1.275
	α-Pinene	0.73	0.523	1.004
	R (+)-Limonene*	0.59	0.415	0.818
	Linalool*	0.474	0.327	0.663
	Eucalyptol*	0.439	0.3	0.617
	(–)-Terpinen-4-ol*	0.422	0.277	0.616
	trans-Cinnamaldehyde*	0.361	0.244	0.508
	Menthone*	0.332	0.217	0.483
	(±)-Citronellal*	0.246	0.156	0.365
	(±)-Camphor*	0.091	0.048	0.156
	Methyl eugenol*	0.137	0.08	0.218
	Bifenthrin	150100.54	14971.335	2574398.3
Geraniol	α-Pinene	0.787	0.562	1.095
	R (+)-Limonene*	0.636	0.449	0.887
	Linalool*	0.511	0.356	0.714
	Eucalyptol*	0.473	0.327	0.664
	(–)-Terpinen-4-ol*	0.455	0.303	0.661
	trans-Cinnamaldehyde*	0.389	0.268	0.543
	Menthone*	0.358	0.238	0.517
	(±)-Citronellal*	0.265	0.172	0.388
	(±)-Camphor*	0.098	0.054	0.164
	Methyl eugenol*	0.148	0.088	0.23
	Bifenthrin*	161726.72	15431.96	2928808.8
α-Pinene	R (+)-Limonene	0.808	0.576	1.123
	Linalool*	0.649	0.459	0.899
	Eucalyptol*	0.601	0.422	0.836
	(–)-Terpinen-4-ol*	0.578	0.391	0.832
	trans-Cinnamaldehyde*	0.494	0.347	0.681
	Menthone*	0.454	0.308	0.649
	(±)-Citronellal*	0.337	0.224	0.485

	(±)-Camphor*	0.124	0.07	0.205
	Methyl eugenol*	0.188	0.115	0.288
	Bifenthrin*	205507.34	19101.794	3844703.4
R (+)-Limonene	Linalool	0.803	0.576	1.107
	Eucalyptol	0.743	0.53	1.029
	(–)-Terpinen-4-ol	0.715	0.491	1.022
	trans-Cinnamaldehyde*	0.612	0.439	0.832
	Menthone*	0.562	0.389	0.793
	(±)-Citronellal*	0.417	0.284	0.59
	(±)-Camphor*	0.154	0.089	0.248
	Methyl eugenol*	0.232	0.147	0.348
	Bifenthrin*	254236.23	22750.683	4985156.3
Linalool	Eucalyptol	0.925	0.674	1.269
	(–)-Terpinen-4-ol	0.89	0.627	1.257
	trans-Cinnamaldehyde	0.761	0.564	1.017
	Menthone*	0.7	0.498	0.971
	(±)-Citronellal*	0.519	0.367	0.717
	(±)-Camphor*	0.191	0.116	0.298
	Methyl eugenol*	0.289	0.191	0.42
	Bifenthrin*	316592.76	27008.329	6588016.8
Eucalyptol	(–)-Terpinen-4-ol	0.962	0.675	1.365
	trans-Cinnamaldehyde	0.823	0.607	1.104
	Menthone	0.757	0.537	1.053
	(±)-Citronellal*	0.561	0.396	0.778
	(±)-Camphor*	0.207	0.125	0.323
	Methyl eugenol*	0.312	0.206	0.455
	Bifenthrin*	342200.21	29059.56	7160424.7
(–)-Terpinen-4-ol	trans-Cinnamaldehyde	0.855	0.615	1.184
	Menthone	0.786	0.545	1.126
	(±)-Citronellal*	0.583	0.404	0.828
	(±)-Camphor*	0.215	0.13	0.339
	Methyl eugenol*	0.325	0.212	0.48
	Bifenthrin*	355549.9	29407.712	7679913.1
trans-Cinnamaldehyde	Menthone	0.919	0.676	1.248
	(±)-Citronellal*	0.682	0.503	0.913
	(±)-Camphor*	0.251	0.16	0.377
	Methyl eugenol*	0.38	0.263	0.531
	Bifenthrin*	415757.78	33831.377	9177737.7

Menthone	(±)-Citronellal	0.742	0.528	1.031
	(±)-Camphor*	0.273	0.17	0.42
	Methyl eugenol*	0.413	0.278	0.595
	Bifenthrin*	452261.46	36449.519	10093068
(±)-Citronellal	(±)-Camphor*	0.369	0.238	0.553
	Methyl eugenol*	0.557	0.387	0.786
	Bifenthrin*	609884.82	47124.797	14343916
(±)-Camphor	Methyl eugenol*	1.511	1.001	2.311
	Bifenthrin*	1654624.3	110713.93	46402083
Methyl eugenol	Bifenthrin*	1095413	78031.01	28471929

Supplementary Table S2. Relative median potency comparisons for topical LD_{50} estimates for essential oil components and bifenthrin. Asterisks (*) indicate components which show significantly higher or lower toxicity in comparison to the baseline essential oil components or insecticides. The LD_{50} estimates for components or insecticides are significantly different ($P < 0.05$) from the baseline compounds if confidence intervals for median potency ratios do not overlap with the value 1 (References 66 and 67). [I] Relative median potency ratios of < 1 indicate essential oil components or insecticides that are less toxic than the baseline compounds they are compared with, whereas ratios >1 indicate comparatively higher toxicity (References 66 and 67).

**Baseline essential oil components or insecticides [(I)]**	**Essential oil components or insecticides for comparison [(I)]**	**Relative median potency ratios [(II)]**	**Confidence intervals (CIs)**	
			**Lower limit**	**Upper limit**
Thymol	Linalool*	0.430	0.195	0.810
	Carvacrol*	0.397	0.165	0.786
	(±)-Camphor*	0.152	0.039	0.393
	Menthone*	0.133	0.035	0.332
	Eucalyptol*	0.123	0.031	0.310
	(–)-Terpinen-4-ol*	0.047	0.007	0.162
	trans-Cinnamaldehdye*	0.055	0.009	0.177
	R (+)-Limonene*	0.041	0.006	0.142
	α-Pinene*	0.050	0.008	0.168
	(±)-Citronellal*	0.018	0.000	0.128
	DDVP*	445.222	44.138	16552.345
Linalool	Carvacrol	0.924	0.487	1.683
	(±)-Camphor*	0.353	0.128	0.752
	Menthone*	0.309	0.118	0.621
	Eucalyptol*	0.286	0.107	0.573
	(–)-Terpinen-4-ol*	0.110	0.025	0.294
	trans-Cinnamaldehdye*	0.127	0.033	0.322
	R (+)-Limonene*	0.096	0.022	0.256
	α-Pinene*	0.116	0.027	0.301
	(±)-Citronellal*	0.042	0.002	0.214
	DDVP*	1034.877	79.023	58505.699
Carvacrol	(±)-Camphor*	0.382	0.147	0.803
	Menthone*	0.335	0.136	0.659
	Eucalyptol*	0.310	0.124	0.606
	(–)-Terpinen-4-ol*	0.119	0.030	0.305
	trans-Cinnamaldehdye*	0.138	0.038	0.335
	R (+)-Limonene*	0.104	0.026	0.265
	α-Pinene*	0.125	0.032	0.312
	(±)-Citronellal*	0.064	0.012	0.193
	DDVP*	1120.562	79.514	70970.060
(±)-Camphor	Menthone	0.877	0.442	1.718
	Eucalyptol	0.811	0.416	1.531
	(–)-Terpinen-4-ol*	0.311	0.118	0.658
	trans-Cinnamaldehdye*	0.361	0.147	0.741

	R (+)-Limonene*	0.272	0.104	0.560
	α-Pinene*	0.328	0.130	0.658
	(±)-Citronellal*	0.116	0.015	0.480
	DDVP*	2935.675	143.366	334383.574
Menthone	Eucalyptol	0.925	0.532	1.578
	(–)-Terpinen-4-ol*	0.355	0.148	0.689
	trans-Cinnamaldehdye*	0.412	0.186	0.773
	R (+)-Limonene*	0.310	0.132	0.583
	α-Pinene*	0.375	0.165	0.684
	(±)-Citronellal*	0.134	0.019	0.421
	DDVP*	3348.451	162.751	387920.248
Eucalyptol	(–)-Terpinen-4-ol*	0.383	0.171	0.711
	trans-Cinnamaldehdye*	0.445	0.214	0.800
	R (+)-Limonene*	0.336	0.153	0.600
	α-Pinene*	0.405	0.191	0.702
	(±)-Citronellal*	0.144	0.023	0.427
	DDVP*	3618.574	170.936	439980.384
(–)-Terpinen-4-ol	trans-Cinnamaldehdye	1.162	0.625	2.252
	R (+)-Limonene	0.875	0.487	1.547
	α-Pinene	1.056	0.605	1.828
	(±)-Citronellal*	0.378	0.105	0.944
	DDVP*	9438.613	319.082	1939854.938
trans-Cinnamaldehdye	R (+)-Limonene	0.753	0.406	1.317
	α-Pinene	0.909	0.507	1.551
	(±)-Citronellal*	0.327	0.08	0.84
	DDVP*	8123.118	294.933	1491225.139
R (+)-Limonene	α-Pinene	1.206	0.749	1.960
	(±)-Citronellal*	0.435	0.137	0.991
	DDVP*	10784.277	357.170	2301043.075
α-Pinene	(±)-Citronellal*	0.355	0.112	0.790
	DDVP*	8939.013	301.710	1854427.110
(±)-Citronellal	DDVP*	17610.837	503.306	4696063.162

Supplementary Table S3. Relative median potency comparisons for fumigant LC_{50} estimates for essential oil components and bifenthrin. Asterisks (*) indicate components which show significantly higher or lower toxicity in comparison to the baseline essential oil components or insecticides. The LC_{50} estimates for components or insecticides are significantly different ($P < 0.05$) from the baseline compounds if confidence intervals for median potency ratios do not overlap with the value 1 (References 66 and 67). [I] Relative median potency ratios

of < 1 indicate essential oil components or insecticides that are less toxic than the baseline compounds they are compared with, whereas ratios >1 indicate comparatively higher toxicity (References 66 and 67).

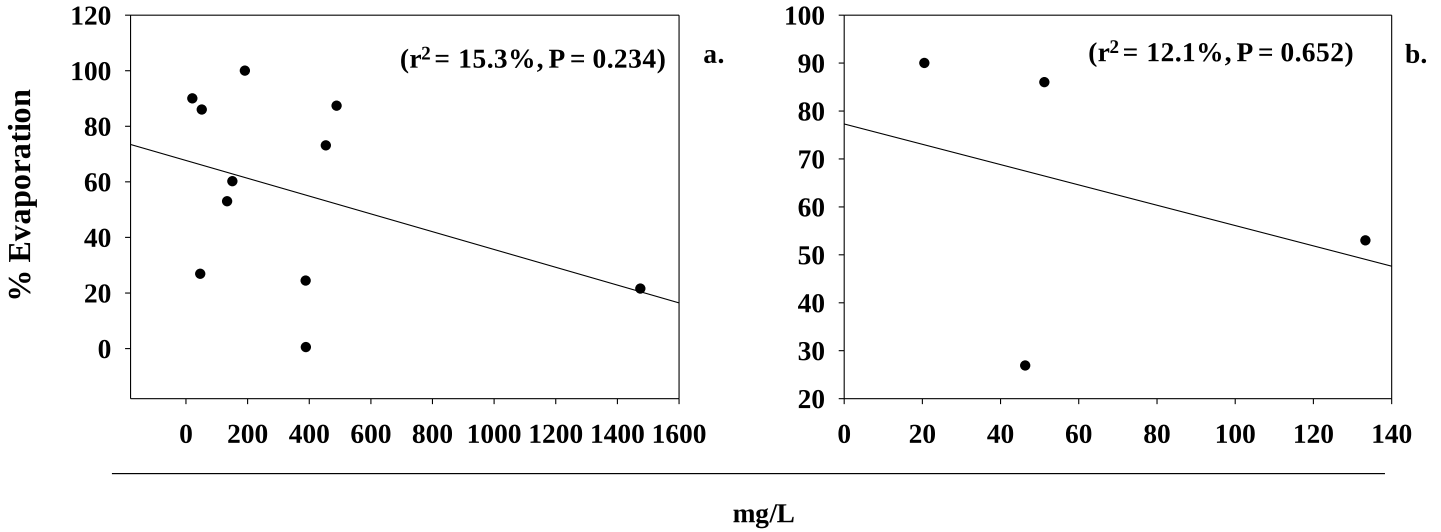

Figure S1. Regression analysis of fumigant LC_{50} values for essential oil components and their corresponding percent evaporation levels. Correlation coefficients (r^2) and P-values were determined by regression analysis. **(a)** Regression analysis for 11 compounds for which we were able to determine LC_{50} values as shown in Table 2. There was no significant correlation between fumigant LC_{50} values and percent evaporation levels ($P > 0.05$). The least toxic compounds (geraniol, citronellic acid, eugenol and methyl eugenol) were excluded from this analysis because their LC_{50} estimates were not determinable. **(b)** Regression analysis for the four most toxic fumigant compounds shown in Table 2 (thymol, carvacrol, linalool, and (±)-camphor. There was no significant correlation between fumigant LC_{50} values of these four most toxic compounds and their percent evaporation data ($P > 0.05$).

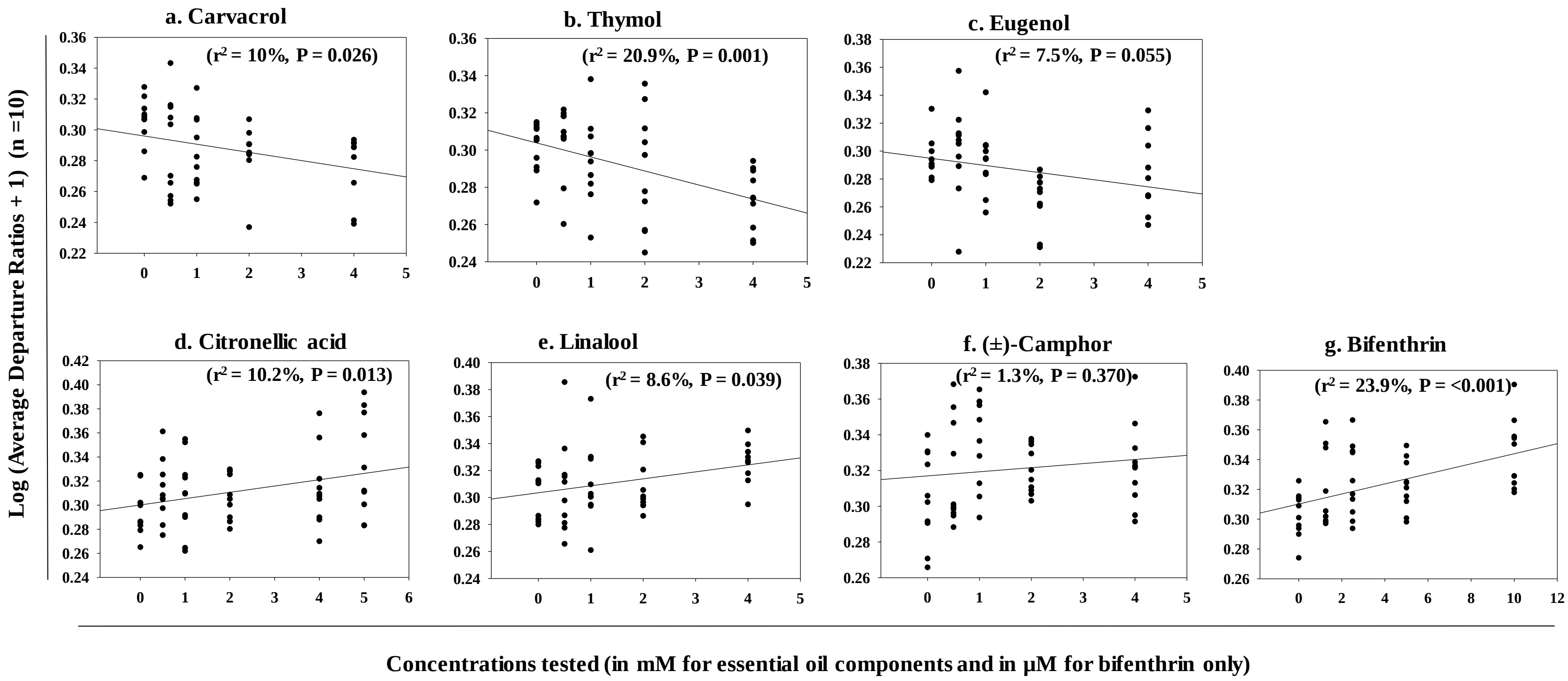

Figure S2. Regression analysis between concentrations of essential oil components or bifenthrin versus log transformed average departure ratio data from electrophysiology experiments. Correlation coefficients (r^2) and P-values were determined by regression analysis. There was significant correlation between concentrations of carvacrol (a), thymol (b), citronellic acid (d), linalool (e) and bifenthrin (f) and their log transformed average departure ratio data ($P < 0.05$), thus indicating concentration-dependent effect of these compounds on the bed bug nervous system. However, eugenol (c) and (±)-camphor (f) concentrations were not correlated with log transformed average departure ratios ($P > 0.05$), likely because of their effects on the nervous system activity were biphasic i.e. pronounced effect at intermediate concentrations in comparison to lower or higher concentrations.
